# Giant Mesothelial Cyst of the Spermatic Cord: A Report of a Rare Case

**DOI:** 10.7759/cureus.69898

**Published:** 2024-09-22

**Authors:** Ramki Arunachalam Ganesh, Jesu Pencilin Yesuvadiyan, Karthikeyan Selvaraj

**Affiliations:** 1 General Surgery, Sree Balaji Medical College and Hospital, Chennai, IND

**Keywords:** extratesticular cyst, mesothelial cyst, mesothelial cyst of spermatic cord, spermatic cord cyst, spermatocele

## Abstract

Mesothelial cysts are uncommon benign lesions formed by mesothelial cells that line the serosal surfaces of many organs. It is commonly found in the peritoneum and less commonly in the pleura; when found in the spermatic cord, they are exceedingly rare. These cysts usually appear as painless lumps in the inguinal or scrotal area and often remain asymptomatic. Here, we describe a 64-year-old male who presented with scrotal swelling for four years and was clinically diagnosed with a spermatocele. He was taken up for an excision biopsy. The histopathology of the cyst was consistent with a mesothelial cyst. Herein, we discuss the clinical presentation, diagnostic approach, and management with a review of the literature on this uncommon entity.

## Introduction

The mesothelial cyst of the spermatic cord is a benign lesion that originates from the mesothelial cells lining the peritoneum [1]. Mesothelial cysts are more commonly found in the peritoneum and less commonly in the pleura and rarely in other locations such as the spermatic cord. Spermatic cord cysts are usually found in less than 1% of males during the dissection of the inguinal hernia. Mesothelial cysts of the spermatic cord are extremely rare, with only a handful of cases reported in the literature. These cysts often present as painless inguinal or scrotal swellings. Despite their benign nature, they can be difficult to diagnose since they seem similar to other cystic diseases of the spermatic cord, such as epididymal cysts, spermatoceles, and hydroceles [2]. Accurate pre-operative diagnosis is challenging, and definitive diagnosis often requires histopathological examination (HPE) following surgical excision [3]. Here, we present the case of a mesothelial cyst of the spermatic cord and review the existing literature on this topic.

## Case presentation

A 64-year-old male presented with complaints of painless swelling over the right side of his scrotum for the past four years, which was insidious in onset, was initially small in size, but gradually increased to attain its current size. The patient gives a history of discomfort due to the size of the swelling. There was no prior history of trauma. Swelling did not increase in size with coughing and did not decrease in size on lying supine. There was no prior history of similar swelling. The patient has a known history of coronary artery disease and post-coronary artery bypass grafting (CABG) done in 2015. There were no other known co-morbidities.

**Figure 1:**
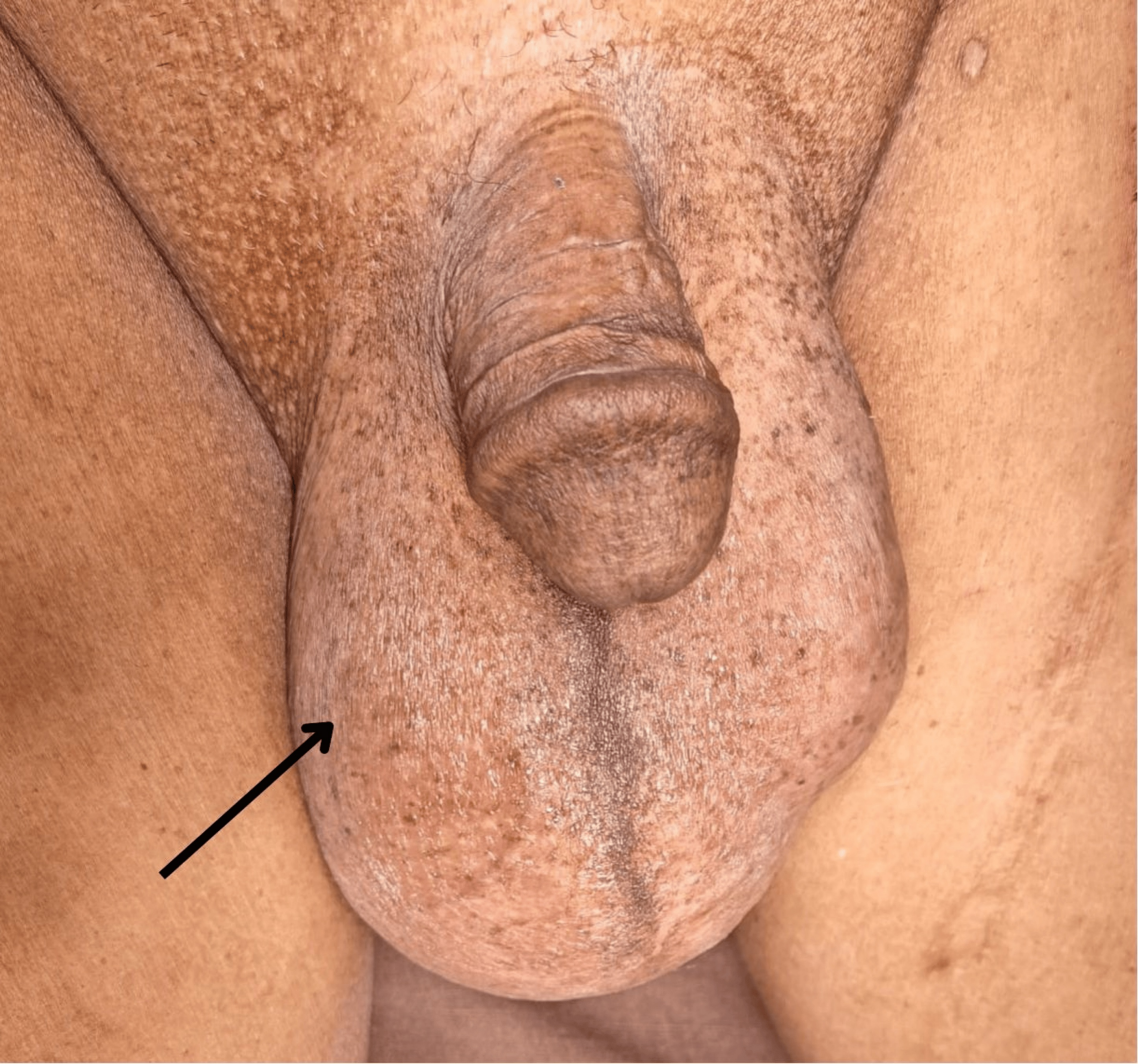
Clinical picture of the swelling occupying the right hemiscrotum (marked with black arrow)

Physical examination revealed a swelling of size approximately 10 × 8 cm (Figure 1), which was confined to the right hemiscrotum, superior and anterior to the testis. The testis was palpable separately. The swelling was cystic in consistency and fluctuant. There was no cough impulse noted. The transillumination test was positive.

**Figure 2:**
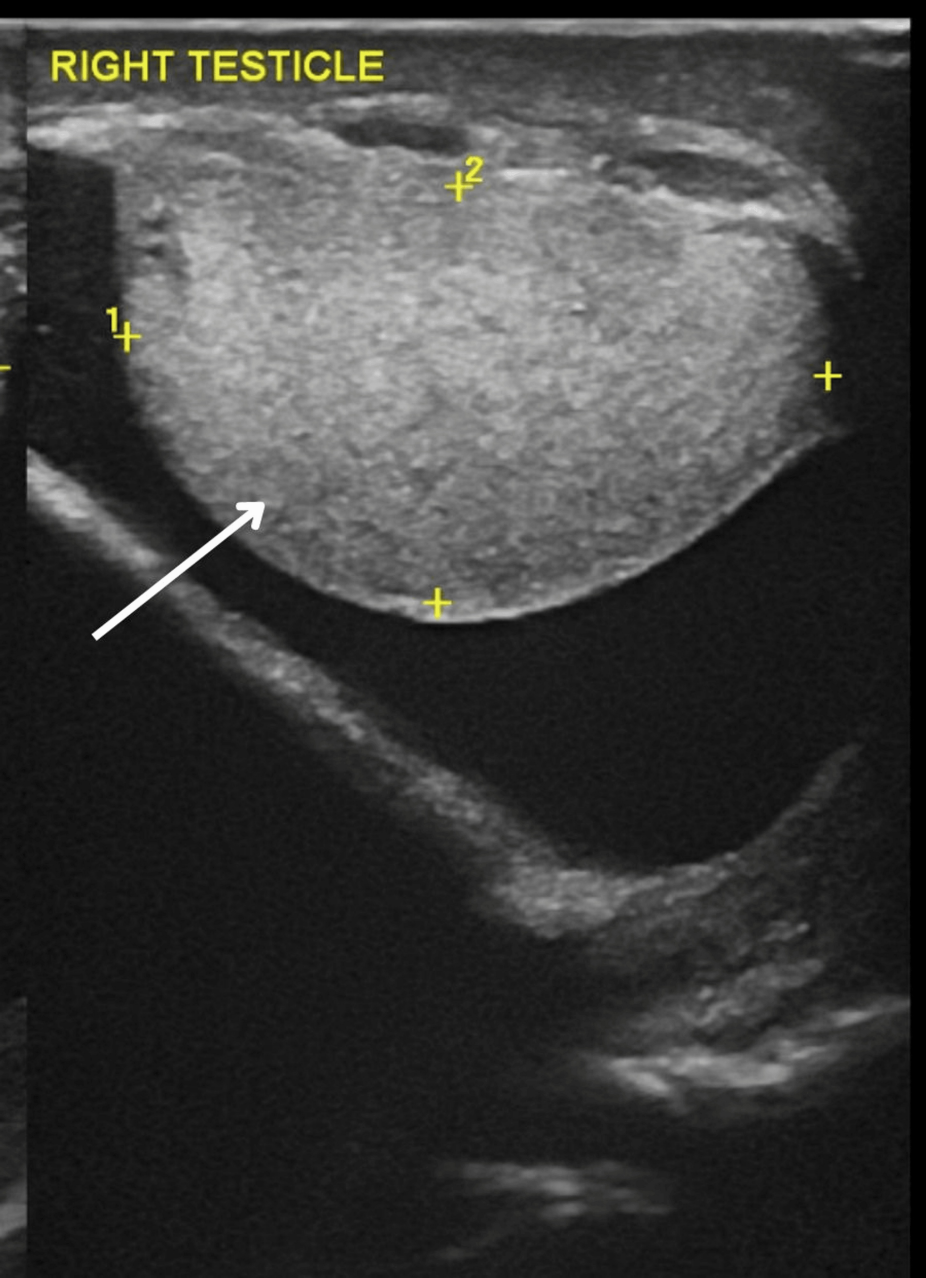
Ultrasound of the scrotum showing the right testis (marked with white arrow)

**Figure 3:**
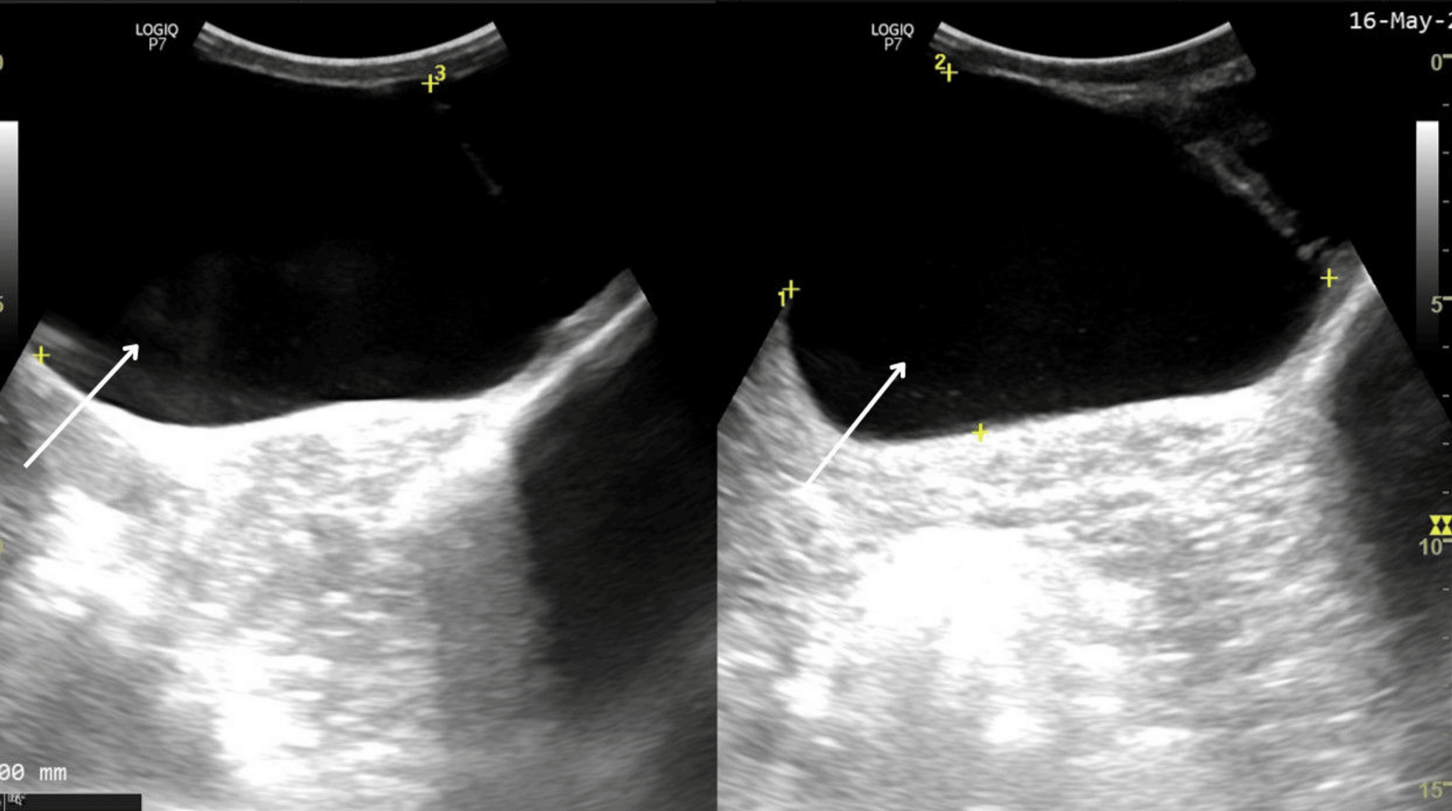
Ultrasound showing the cyst (marked with white arrow)

His scrotal ultrasound revealed a large cystic lesion of size 11 × 9.7 × 7.4 cm in the spermatic cord superior to the testis within the right scrotum (Figure 2 and Figure 3).

The patient's blood and urine investigations done were found to be normal. He was clinically diagnosed with spermatocele. The patient underwent surgical exploration and excision of the cyst.

**Figure 4:**
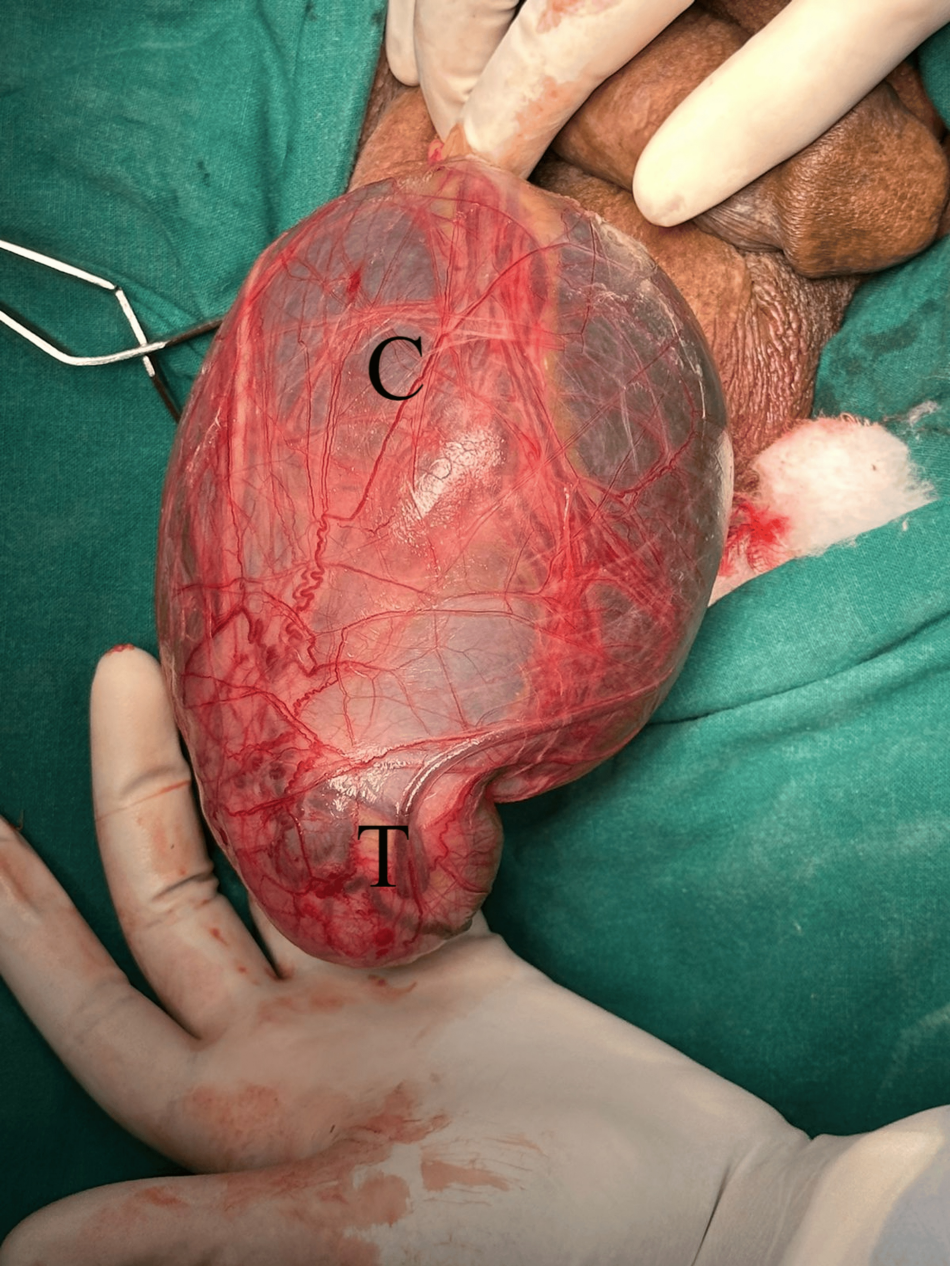
Intra-operative picture showing the cyst (C) within the cord structures and testis (T)

**Figure 5:**
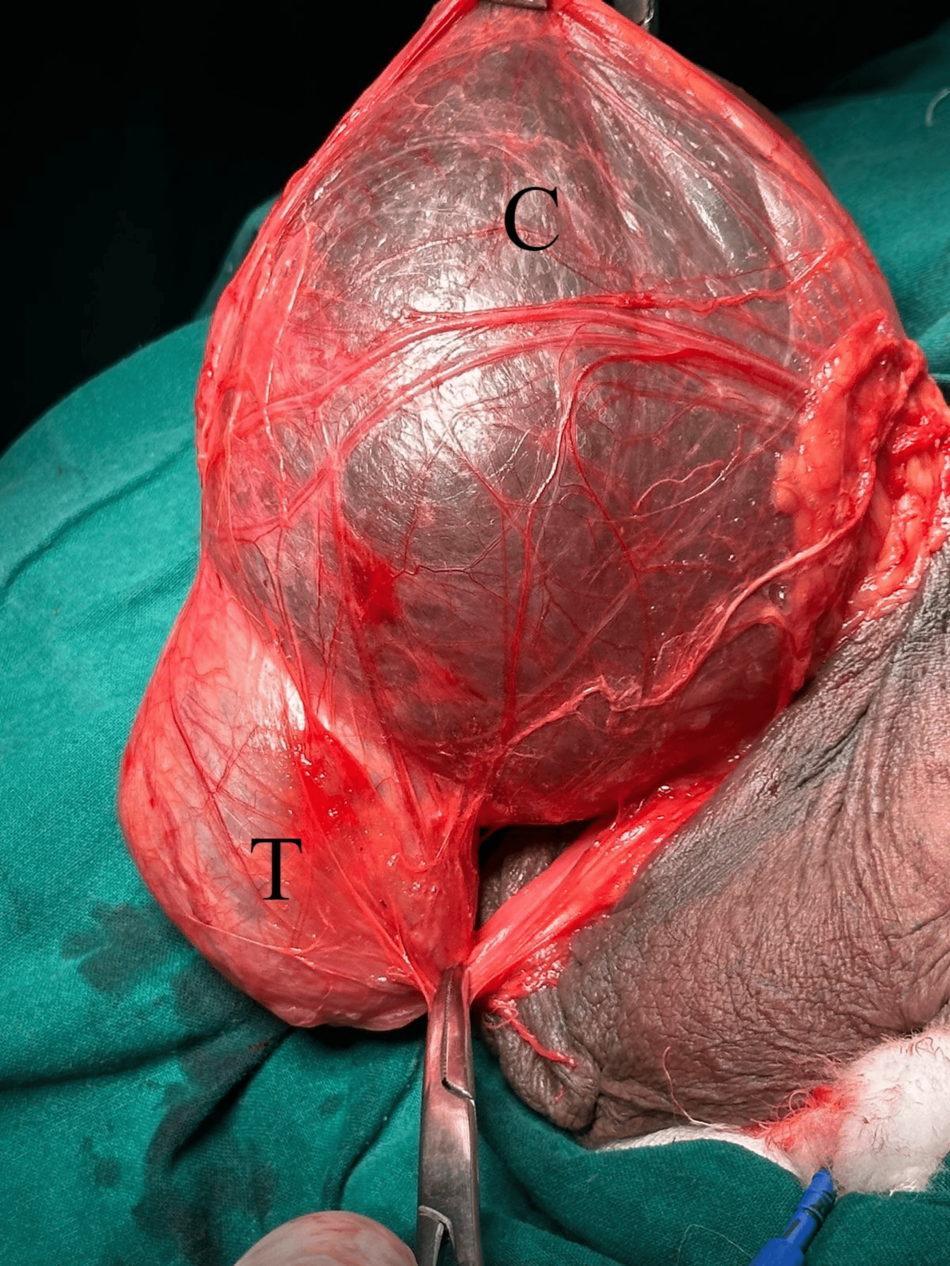
Intra-operative picture showing the cyst (C) within the cord structures and testis (T)

**Figure 6:**
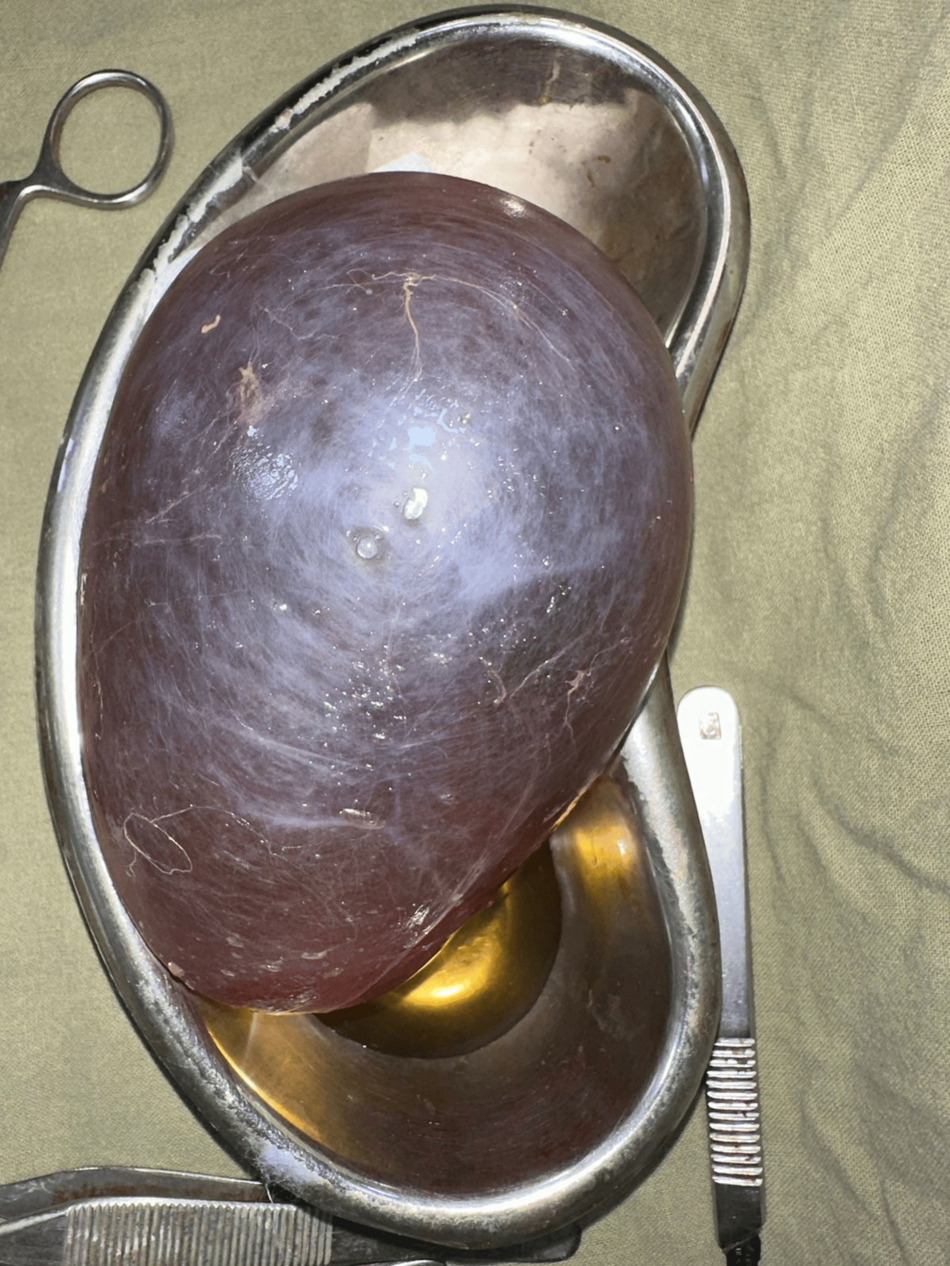
Specimen of the excised cyst

Through paramedian raphe incision, the cyst along with the right testis and cord structures were delivered out. A unilocular, barley water-colored fluid-filled cyst of size approximately 12 × 10 × 9 cm was excised from within the cord structure, which was found to be avascular, non-adherent to the vas deferens or spermatic vessels, but adherent to the epididymal head, preserving the epididymis (Figure 4 and Figure 5). The cord structures and the testis were kept in place and the wound was closed in layers. The excised cyst (Figure 6) was then sent for HPE.

Fluid from the cyst was sent for microscopic examination and did not show any spermatozoa.

**Figure 7:**
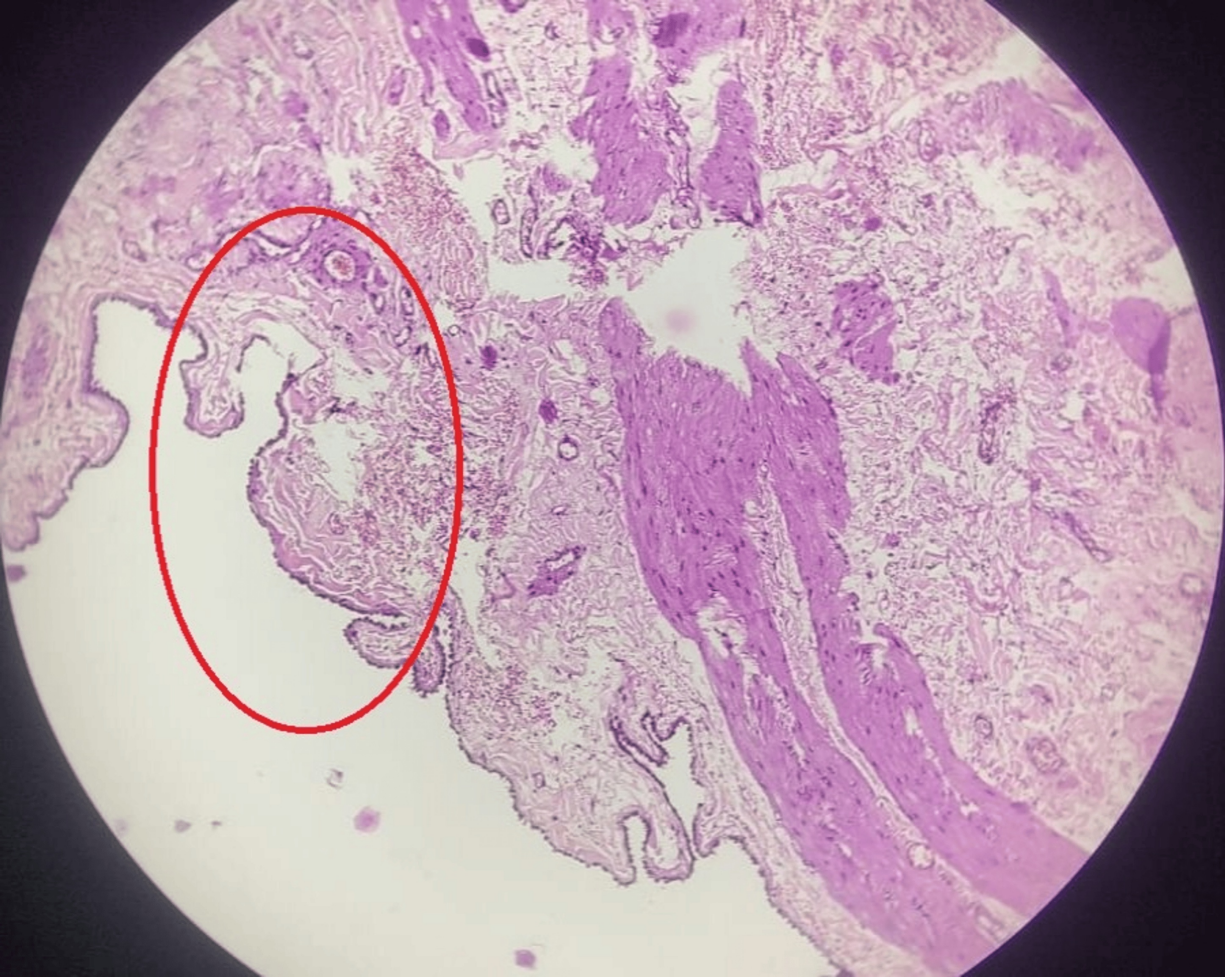
HPE slide showing a low-power view of low cuboidal epithelium with cystic spaces (red circle) HPE: histopathological examination

**Figure 8:**
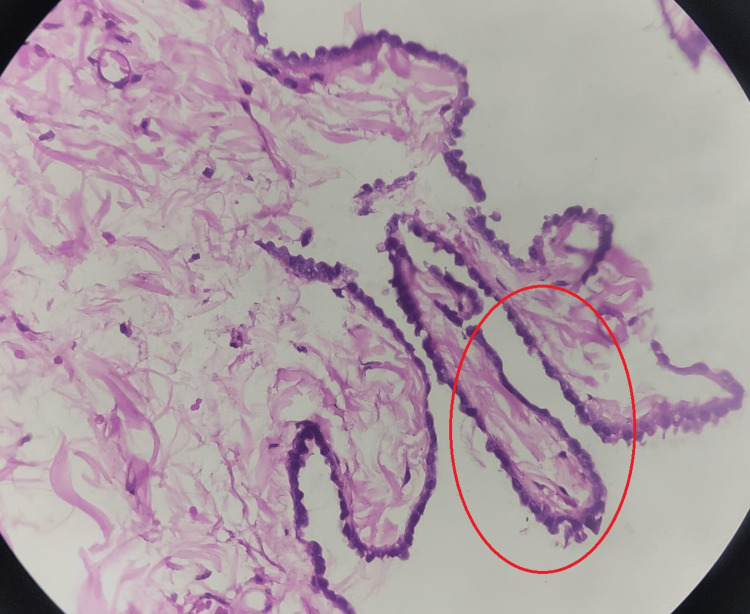
HPE slide showing a high-power view of low cuboidal epithelium with lining thrown into broad papillary-like structures (red circle) HPE: histopathological examination

HPE of the cyst showed a fibromuscular cyst wall lined by low cuboidal epithelium with cystic spaces lined by similar cells (Figure 7) with the lining thrown into broad papillary-like structures (Figure 8) suggestive of a mesothelial cyst.

The post-operative course of the patient was uneventful and had no complications. There was no evidence of cyst recurrence six months post-surgery.

## Discussion

Mesothelial cysts are uncommon benign lesions formed by mesothelial cells, which often line the serosal surfaces of many organs. When these cysts form in the spermatic cord, they are an extremely rare clinical phenomenon [4]. A comprehensive review of the literature reveals that there is sparse case documentation, often limited to solitary case reports [5-7].

The exact etiology of mesothelial cysts of the spermatic cord remains unclear. It is hypothesized that these cysts arise from mesothelial remnants or embryonic remnants that fail to involute during development. They are typically located within the paratesticular tissues and can vary in size from a few millimeters to several centimeters. Clinically, patients often present with a painless scrotal swelling, similar to other more common cystic lesions of the scrotum such as epididymal cyst, encysted hydroceles, or spermatoceles. It can occur in any age group, but is mostly seen in patients above 40 years of age. In certain instances, a mesothelial cyst of the spermatic cord is also misdiagnosed with undescended testis, inguinal hernia, or mass [5,6,8].

Mesothelial cysts show with a vague clinical picture that makes diagnosis difficult. Imaging techniques such as ultrasonography, computed tomography (CT), or magnetic resonance imaging (MRI) can give useful information regarding the size, location, and cystic nature of the lesion. However, conclusive diagnosis generally needs histological evaluation following surgical excision [7,9,10].

Histologically, mesothelial cysts of the spermatic cord are characterized by a cystic structure lined by a single layer of mesothelial cells. The cyst wall consists of fibrous tissue, and the cystic contents are usually clear serous fluid [11].

Although the location of the cyst in the present case was adherent to the epididymal head which is also the feature of spermatocele or epididymal cyst, the microscopic examination of the fluid content of the cyst did not reveal any spermatozoa. In the case reports by Salama and Hassan and Takimoto et al., they had similar findings of no microscopic evidence of spermatozoa, but they had concluded it to be a spermatocele based on the histopathologic findings, which in their study was ciliated cuboidal epithelium [12,13]. In our case, the epithelium was non-ciliated, low cuboidal epithelium with lining thrown into broad papillary-like structures which is conclusive of a mesothelial cyst.

Treatment of choice for mesothelial cysts of the spermatic cord is complete surgical excision [3,6]. This approach not only provides a definitive diagnosis but also ensures the complete removal of the cyst and usually prevents recurrence. Since there have been no documented cases of malignant transformation of these benign lesions in the literature, the prognosis is excellent.

## Conclusions

Mesothelial cysts of the spermatic cord, although rare, are typically benign lesions that are often discovered incidentally and are usually asymptomatic. Accurate diagnosis is crucial and involves distinguishing these cysts from other similar scrotal lesions, such as epididymal cysts and hydroceles. Diagnostic imaging, primarily ultrasound, helps in identifying the cystic structure, but definitive diagnosis often requires HPE following surgical excision. This approach not only confirms the benign nature of the cyst but also addresses any diagnostic uncertainty.

The post-surgical prognosis for mesothelial cysts is excellent, with a very low risk of recurrence and favorable patient outcomes. Most patients experience a resolution of symptoms and a swift return to normal activities. Effective management and appropriate surgical intervention ensure that patients are reassured and can expect a positive long-term outcome. Continued research and refined diagnostic techniques will further enhance our understanding and treatment of these rare lesions, contributing to improved patient care and management strategies.
